# Anti-Tumor Effects of Bak-Proteoliposomes against Glioblastoma

**DOI:** 10.3390/molecules200915893

**Published:** 2015-09-01

**Authors:** Lavinia Liguori, Fabio Pastorino, Xavier Rousset, Silvia Alfano, Sandra Cortes, Laura Emionite, Antonio Daga, Mirco Ponzoni, Jean-Luc Lenormand

**Affiliations:** 1 SyNaBi Laboratory, TIMC IMAG, UMR S5525, UJF/CNRS, Joseph Fourier University, Grenoble Cedex 9 38700, France; 2 Laboratory of Oncology, Istituto Giannina Gaslini, Genoa 16147, Italy; E-Mails: fabiopastorino@ospedale-gaslini.ge.it (F.P.); mircoponzoni@ospedale-gaslini.ge.it (M.P.); 3 The Rex Laboratory, TIMC IMAG, UMR5525, UJF/CNRS, Joseph Fourier University, CHU-Grenoble, BP217, Grenoble Cedex 9 38043, France; E-Mails: xavier.rousset@gmail.com (X.R.); silvialfn@gmail.com (S.A.); sandra.cortes@synthelis.fr (S.C.); jllenormand@chu-grenoble.fr (J.-L.L.); 4 Animal Facility, IRCCS Azienda Ospedaliera Universitaria San Martino-IST, Genoa 16132, Italy; E-Mail: laura.emionite@hsanmartino.it; 5 Laboratorio di Trasferimento Genico, IRCCS Azienda Ospedaliera Universitaria San Martino-IST, Genoa 16132, Italy; E-Mail: antonio.daga@hsanmartino.it

**Keywords:** recombinant membrane protein, Bak, proteoliposomes, glioblastoma

## Abstract

Despite palliative treatments, glioblastoma (GBM) remains a devastating malignancy with a mean survival of about 15 months after diagnosis. Programmed cell-death is de-regulated in almost all GBM and the re-activation of the mitochondrial apoptotic pathway through exogenous bioactive proteins may represent a powerful therapeutic tool to treat multidrug resistant GBM. We have reported that human Bak protein integrated in Liposomes (LB) was able, *in vitro*, to activate the mitochondrial apoptotic pathway in colon cancer cells. To evaluate the anti-tumor effects of LB on GBM, MTT (3-(4,5-dimethylthiazol-2-yl)-2,5-diphenyltetrazolium bromide) assays and Western blot analysis were performed on GL26 murine cell line. LB treatment shows a dose-dependent inhibition of cell viability, followed by an up-regulation of Bax and a down-modulation of JNK1 proteins. In GL26-bearing mice, two different routes of administration were tested: intra-tumor and intravenous. Biodistribution, tumor growth and animal survival rates were followed. LB show long-lasting tumor accumulation. Moreover, the intra-tumor administration of LB induces tumor growth delay and total tumor regression in about 40% of treated mice, while the intravenous injection leads to a significant increased life span of mice paralleled by an increased tumor cells apoptosis. Our findings are functional to the design of LB with potentiated therapeutic efficacy for GBM.

## 1. Introduction

Despite palliative treatments including surgery, radiotherapy and chemotherapy, the prognosis for patients with glioblastoma (GBM) remains poor with a median survival time of ~15 months. The incidence is approximately 2–3 new cases per 100,000 people per year, [1]. Surgery followed by focal radiotherapy and chemotherapy remains the standard first line treatment [2]. However, GBM is a multidrug resistant tumor and it responds poorly to chemotherapy, due to the late and difficult diagnosis and to the insufficient drug homing in the tumor [3]. The other major limiting factor to the effectiveness of treatments is the presence of the blood–brain barrier (BBB), which does not allow the accumulation of therapeutic drugs in the tumor [4].

In many cancers, programmed cell-death is de-regulated. Deciphering the molecular mechanisms underlying apoptosis resistance in tumors has become crucial in order to identify new “smart drugs” for therapy [5]. Despite potential benefits derived from targeting different pathways in a multi-target setting, the likelihood of achieving long-lasting therapeutic effects for patients with recurrent GBM remains uncertain.

Liposomes are delivery systems recently applied to human therapy against cancer or infectious diseases with clinical evidence of efficacy [6,7,8]. Their most important benefits include (i) protection of the encapsulated “drug” against degradation; (ii) long-lasting pharmacokinetic and biodistribution profiles of the drugs, which allow to enhance accumulation of the active molecule in the tumor; and (iii) reduction of the drug side effects [9,10,11]. In addition, taking advantage of the enhanced permeability and retention (EPR) effect, liposomes tend to passively penetrate into the interstitial spaces of the neoplastic sites, especially in those under hypoxia, or in solid tumors where vessel network is irregular [11]. As an example, Doxil(^®^)/Caelyx(^®^), a polyethylene glycol (PEG) liposomal doxorubicin, is one of the leading approved nanoparticles products used in cancer therapy [8]. Similarly, liposomal anthracycline is currently utilized for the treatment of childhood leukemia and lymphomas [7]. Importantly, because of the disruption of the BBB in high-grade GBM, liposomes can extravasate from blood vessels and deliver their content into the tumor [12].

Mitochondrial membrane perturbation is one of the key events in response to cell-death signals. Bcl-2 proteins family is strongly involved in the modulation of the cellular life/death equilibrium. Some members, such as Bax and Bak or the BH3-only proteins, can induce apoptosis by mitochondrial membrane permeabilization; in contrast, Bcl-2 and Bcl-XL have an inhibitory effect on cellular death [13]. Compared to the other soluble pro-apoptotic members, Bak is anchored to the outer mitochondrial membrane through a C-terminal domain. Upon cytotoxic signals, Bak oligomerizes (Bak/Bak and/or Bax/Bak) and forms pores, leading to the release of apoptogenic proteins (*i.e.*, cytochrome C and Smac/DIABLO) from the inter-membrane space of mitochondria into the cytoplasm, giving the start signal to the intrinsic apoptotic cascade [14].

The use of membrane proteins (MPs) as therapeutic molecules is an attractive perspective to activate apoptosis in cancer. However, the major bottleneck in their utilization is the low yield of production when expressed using classical cellular over-expression systems [15,16]. To overcome this limitation, we optimized a bacterial cell-free expression system. Combining lipid technology with cell-free system, we were able to synthesize recombinant proteoliposomes containing Bak (LB) in a one-step-reaction [17]. *In vitro* experiments on colon cancer cells showed that, after cellular uptake, LB activate the mitochondrial apoptosis pathway [17].

Here, we confirm, *in vitro*, the pro-apoptotic potential of LB extending its efficacy against the murine GL26 glioblastoma cells. Then, a novel preclinical approach, based on using LB as a “membrane protein-drug” was evaluated *in vivo* in a proof-of-principle mouse model of GBM. Intra-tumor injections of LB induce a significant tumor growth delay and a complete tumor regression in more than 40% of treated animals. Furthermore, animals treated intravenously with LB show an increased tumor cells apoptosis and an enhanced life span compared to control mice.

Altogether, the results obtained allow hypothesizing the development of novel therapeutic strategies for GBM based on the use of LB, alone, or in combination with established protocols.

## 2. Results and Discussion

### 2.1. LB Production and Cell Viability

Glioblastoma multiforme (GBM) is the most common malignant primary brain tumor in adults and the current clinical approaches frequently presents incomplete cancer eradication and associated resistance to “drugs” [18,19]. Advances in the understanding of the molecular mechanisms behind cellular carcinogenesis suggest the possibility of utilizing biologically-derived drugs for specific interventions, in order to alter intracellular signaling pathways inducing apoptosis or chemo-sensitization. Aberrant expression of membrane mitochondrial proteins is one of the strongest inputs for cell death and the delivery of mitochondrial pro-apoptotic proteins can represent a valid therapeutic strategy in cancer [20]. The double challenge we faced in the design of the protein-based drug Bak-proteoliposomes (LB) was (i) the choice of an appropriate membrane protein to be used as a therapeutic agent; and (ii) the development of a proper delivery system able to cross cellular membranes of cancer cells.

The bottleneck in Bak production was overcome by utilizing an optimized cell-free expression system adapted for membrane proteins. The real advance in the cell-free technology developed in our laboratory was the one-step production of functional LB with a yield suitable for therapeutic application. To synthesize LB (Bak Liposomes) and LBΔBH3 (BakΔBH3 Liposomes), lipid vesicles were directly added to the RTS mixture at 1:1 *v*/*v* ratio (5 mg/mL lipid final concentration). During the synthesis reaction, the freshly produced proteins spontaneously integrated lipid vesicles and, once purified, Liposomes with integrated Bak and BakΔBH3 were obtained [21].

**Figure 1:**
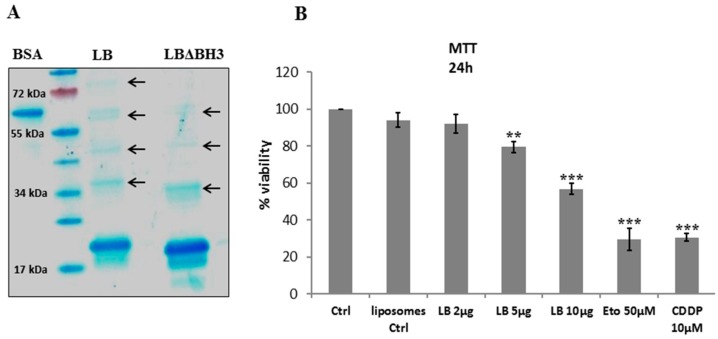
Bak Liposomes (LB) induce cell viability inhibition. (**A**) Coomassie Blue staining of LB and Liposomes with Bak missing the pro-apoptotic domain BH3 (LBΔBH_3_). Bovine serum albumin (BSA) (0.25 mg/mL) is used as reference protein. Arrows indicate protein oligomers; (**B**) MTT viability test on GL26 cells treated for 24 h with increasing doses of LB. Histograms represent the average ± standard deviation (S.D.) of 5 experiments. Ctrl: untreated cells; liposomes Ctrl: cells treated with empty liposomes; Eto: etoposide; CDDP: cisplatin. ** *p* < 0.01 and *** *p* < 0.001 *vs.* Ctrl.

Coomassie blue staining shows the high degree of purity of proteoliposomes (Figure 1A). For these constructs, two bands are present: the first one at 19 kDa, corresponding to recombinant Bak and the second, at 17 kDa, which resulted from a N-terminal deletion during the synthesis process. Interestingly, the fractions containing highly concentrated proteoliposomes revealed the presence of dimers and trimers, a fundamental pre-requisite to exert its apoptotic effect [17]. Electron microscopy on LB showed that the size of Bak vesicles is between 100 and 150 nm [21].

As this study is based on the evidence that therapeutic LB was able to activate the intrinsic apoptotic pathway in colon cancer [17] and to activate caspase 9 in GL26 cells [21], we first tested LB effectiveness on the viability of GL26 tumor cells. Once LB was added to the culture medium, cells were incubated for 5 h and viability was monitored by a MTT test. The assay shows a statistically significant, dose-dependent cellular death in glioblastoma cells with about 50% viability in presence of 10 µg LB/million of cells (Figure 1B). The negative control treatment, empty liposomes, does not affect cellular viability, while Eto and CDDP induce between 70% and 80% cellular death.

### 2.2. Western Blotting on GL26 Cells

Bak belongs to the BH multi-domain pro-apoptotic members of the Bcl-2 family, which are key proteins in balancing cell survival and apoptosis [14]. Lysates from GL26 cells treated with LB were evaluated by Western blot to identify molecular partners involved in LB-mediated cell death mechanism. U0126, bortezomib (BZM), etoposide (Eto) and Staurosporine (stauro) were used as positive controls. Indeed, U0126 is a selective inhibitor of MEK1/2 acting on ERK 1/2 activation [22], BZM is a specific 26S proteasome inhibitor, currently employed in clinical trials for treatment of different solid tumors [22,23]; Eto is known to act on DNA integrity [24] and stauro induces apoptosis by both caspase-dependent and -independent pathways [25].

The mitogen-activated protein kinases, JNKs and ERK, are instrumental in a large number of cellular responses including apoptosis, and their dysfunction may contribute to tumor formation and progression. In most cases, MEK/ERK pathway plays an anti-apoptotic role by promoting the degradation of pro-apoptotic factors [26]; on the contrary, the role of JNKs pathway in regulating apoptosis is more controversial [27].

**Figure 2:**
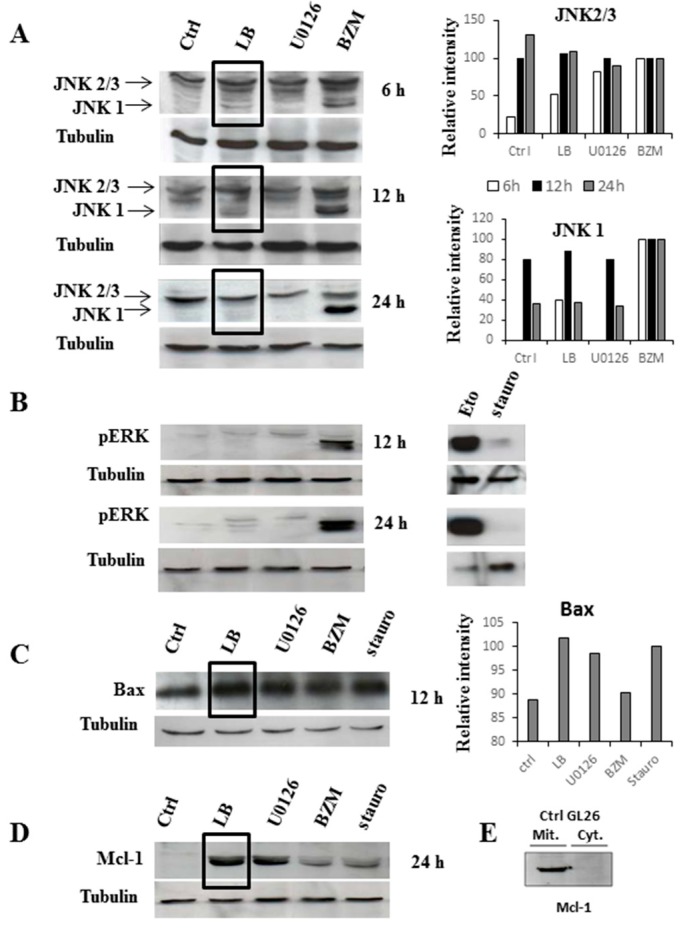
Western Blotting analysis reveals the molecular signaling activated on glioblastoma cells by LB treatment. GL26 cells were treated as described in M & M and lysate at different time points. Proteins (50 µg/lane) were separated in SDS-polyacrylamide gels and transferred to nitrocellulose membranes before incubation with antibodies anti-JNKs, pERK, Bax and Mcl-1. Tubulin antibody was used as internal control for loading. Ctrl: untreated cells; LB: Bak Liposomes; BZM: Bortezomib; Eto: Etoposide; Stauro: Staurosporine. Panels (**A**–**C**): total proteins; panel (**D**): cytoplasmic fraction; and panel (**E**): mitochondrial fraction. Histograms represent the band intensity values; BZM and Stauro treatments are considered as protein-induction positive controls for JNKs and Bax proteins, respectively. Bands analysis was performed using the free software “gel analyzer”. A representative histogram from one out of three experiments (with similar results obtained) is shown.

Here, a constitutive activation of JNK2 and JNK3 and a non-phosphorylated status of ERK is seen in untreated GL26 cells (Figure 2A,B). These results are in agreement with previous data reporting a constitutive JNKs activation in malignant GBM, as a result of EGFR up-regulation [28] and an inhibition of ERK pathway due to mutations of PTEN [29].

JNK1, which is the unique isoform, not constitutively activated, in GL26 cells is affected by exogenous Bak. Although the BZM-induced activation of JNK-1 increased over time (6–24 h), an inverse trend with a maximum activation at 6–12 h is seen after LB treatment (Figure 2A).

It has been reported that MEKK1 seems to control the conformational activation of Bak, leading to formation of the 80–170 kDa Bak complexes correlating with apoptosis. However, JNK1, even if not required for Bak activation, is involved in the stimulation of previously activated Bak to form apoptosis associated complexes [30]. Our results are in agreement with that report, whereby we can suppose that JNK1 is stimulated after LB incubation to activate the endogenous-exogenous Bak complexes. At 24 h treatment, JNK1 activity most likely decreases as the consequence of exogenous Bak accumulation and the unbalanced apoptotic signals. However, further studies are needed to elucidate this process at molecular level.

LB clearly do not affect ERK1/2 phosphorylation, while BZM and Eto elicit a time-dependent and sustained ERK1/2 activation (Figure 2B), as previously reported [31].

We then investigated Bax (pro-apoptotic) and Mcl-1 (anti-apoptotic) proteins, belonging to the Bcl-2 family protein. A significant increase on Bax expression is detected 12 h after LB treatment (Figure 2C). This effect is similar to that obtained by treatment with stauro, which is known to activate Bax and the mitochondrial caspase-dependent apoptotic pathway [20,25]. Over-expression of Bax and Bak induced by apoptotic stimuli has already been described [32]. Mcl-1 is located, under physiological conditions, in the mitochondria, as shown for GL26 cells in Figure 2E, and it usually binds to endogenous Bak [33]. Since the half-life of “free” Mcl-1 is relatively short, in the order of 2–3 h [34], we might justify the presence of the observed protein in the cytosolic fraction (Figure 2D) by supposing an interaction of Mcl-1 with LB. The cytosolic Mcl-1 accumulation is also induced by BZM. In fact, BZM blocks the proteasome 26S and inhibits protein degradation [35]. Finally, LB treatment produces neither a variation in the cyclin-dependent kinase activity nor a modification in the expression of nuclear factor-kappa B in glioblastoma tumors (data not shown).

Our results indicate that LB signaling seems to involve JNK1, Mcl-1 and Bax over-expression. From these indications, a two-way mode of action for LB might be suggested. On one hand, exogenous Bak oligomerizes at the mitochondrial membrane, leading to the activation of the caspases’ cascade. On the other hand, the “cytosolic” LB could sequester the anti-apoptotic Mcl-1 protein from endogenous Bak at the outer mitochondrial membrane [33]. Our experimental evidence suggests that a cytosolic increased of Mcl-1 protein after LB treatment might move the equilibrium towards cell death. This intriguing hypothesis needs to be further investigated but the evidence we present in this study can be read as a tangible clue that supports the concept of LB as a therapeutic apoptotic “bullet”.

### 2.3. Biodistribution and Anti-Tumor Effects of LB

Moving from *in vitro* experiments, a syngeneic, proof-of-principle, animal model of GBM was used by injecting GL26 cells in the flank of mice. A therapeutic approach based on two different routes of administration was performed: LB was intra-tumor (*i.t.*; Group A) or intravenous (*i.v.*; Group B) injected and its therapeutic efficacy was evaluated by following the tumor growth kinetics and the overall animal survival. Biodistribution was investigated using SPECT/CT imaging. Currently, this system is employed to create hybrid-imaging systems that overcome the low resolution of nuclear imaging and gives the way to follow the distribution of molecular tracers uptake in detail.

**Figure 3:**
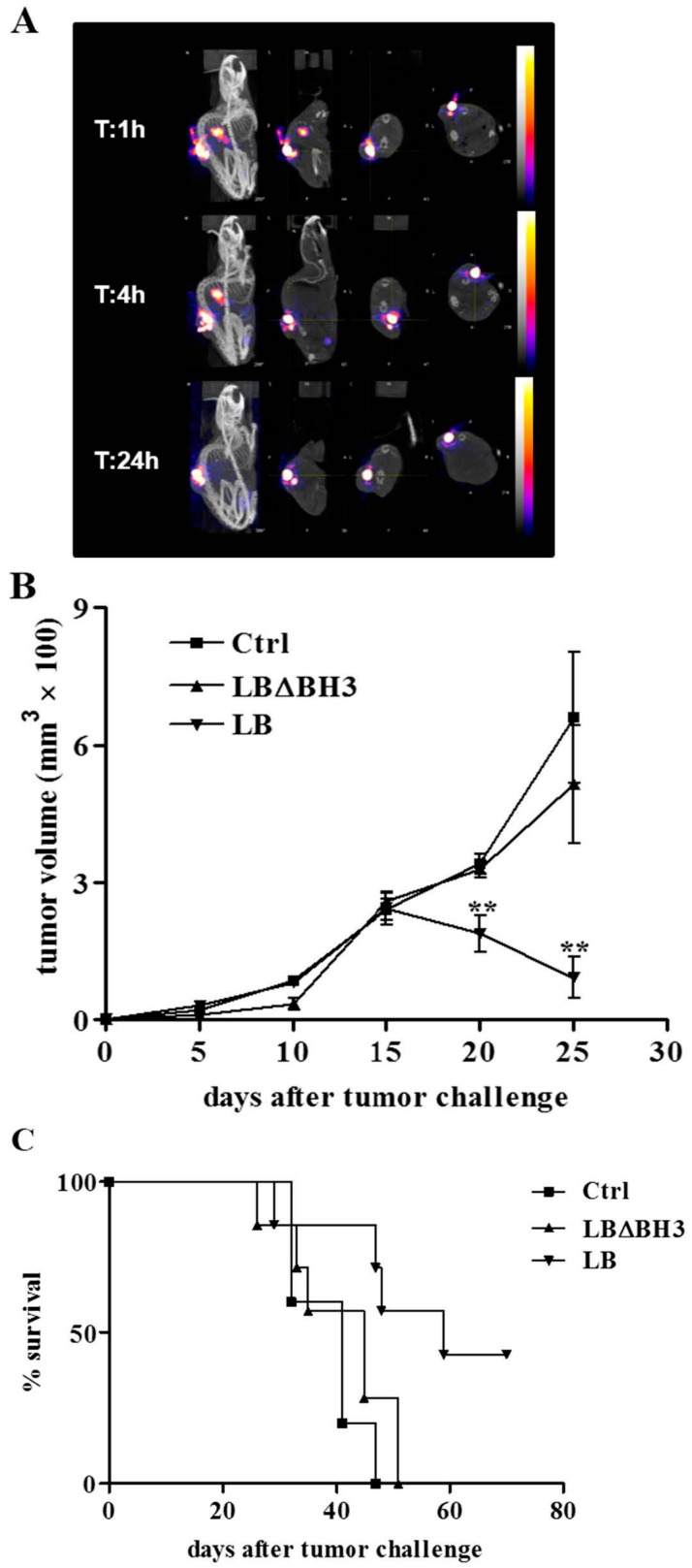
Intra-tumor treatment with LB in GL26-bearing mice. (**A**) SPECT/CT images at 1, 4 and 24 h after treatment with a single intra-tumor injection of ^99m^Tc-LB; (**B**) GL26 tumor growth over time. ** *p* < 0.01, LB *vs.* Ctrl and LBΔBH3; (**C**) Survival curves. *p* < 0.05, LB *vs.* Ctrl and LBΔBH3.

**Figure 4:**
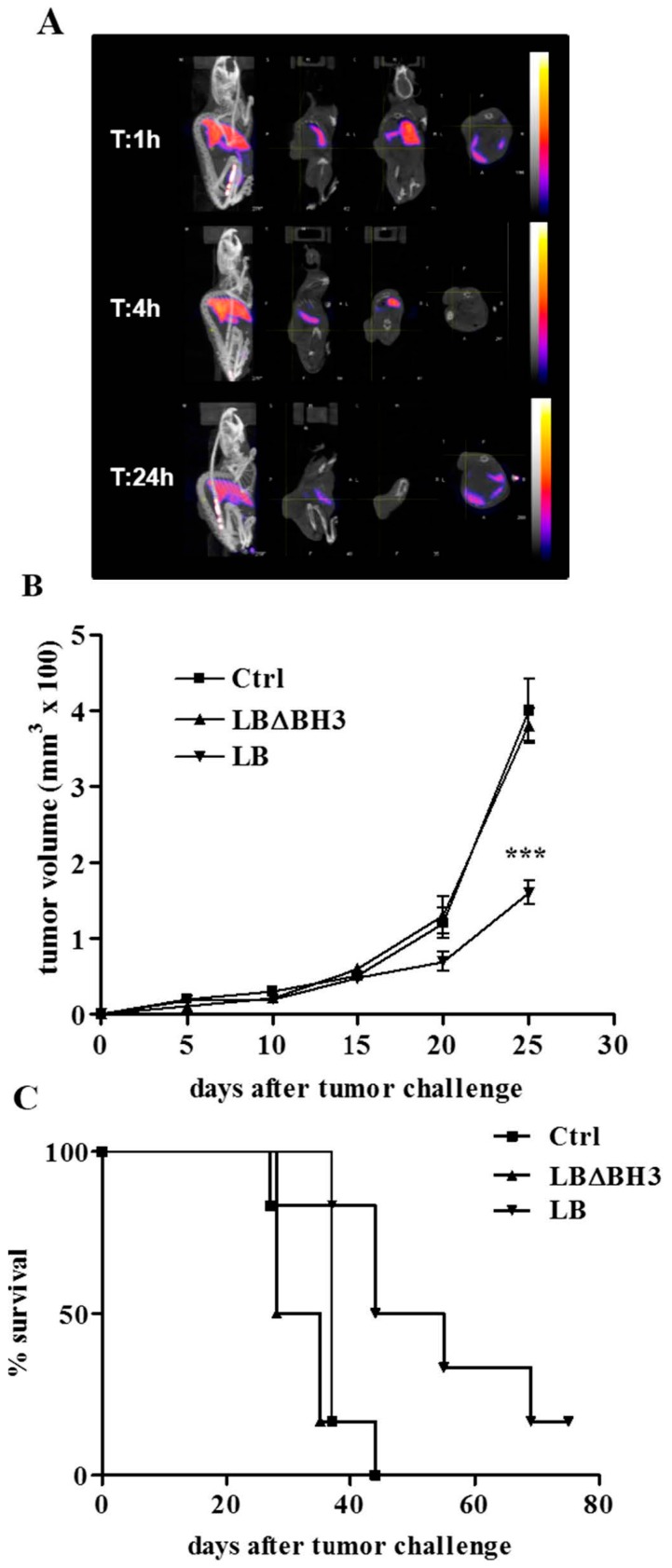
Intravenous treatment with LB in GL26-bearing mice. (**A**) SPECT/CT images at 1, 4 and 24 h after treatment with a single intravenous injection of ^99m^Tc-LB; (**B**) GL26 tumor growth over time. *** *p* < 0.001, LB *vs.* Ctrl and LBΔBH3; (**C**) Survival curves, *p* < 0.05, LB *vs.* Ctrl and LBΔBH3.

In Figure 3 and Figure 4, for each time analyzed (1, 4, 24 h), panels show planes of the animal at various depths acquired during the scan process. In Group A, LB biodistribution indicates that the proteoliposomes, injected directly in the tumor, remain mostly in the mass up to 24 h after treatment (Figure 3A). Moreover, ^99m^Tc radioactivity was measured in all tissues 24 h after LB injection. At this time, LB are still mostly entrapped in the tumor, with non-significant diffusion into the rest of the body (Table 1). The results obtained demonstrate that radioactive-labeled LB injected intra-tumor, remained in the neoplastic mass up to 24 h after administration.

LB biodistribution was then analyzed in Group B, where GL26-bearing received LB in the tail vein. This analysis was performed 4 h after the treatment, taking into account the LB degradation by the clearance process. Similar to other liposomal formulations [36,37], SPECT/CT results indicate a strong accumulation of LB in the liver, spleen lung and urine (Figure 4A and Table 1).

In Group A, measurements have been done 24 h after LB injection. In Group B, measurements have been done 4 h after LB injection. Data are expressed as mean of the % ID/g of tissue ± S.D.

**Table 1:** ^99m^Tc radioactivity in organs after intra-tumor or intravenous injections in mice.

Organ	Group A Intra-Tumor Injection	Group B Intravenous Injection
Brain	0.005 ± 0.009	0.030 ± 0.006
Heart	0.004 ± 0.003	0.282 ± 0.057
Stomach	0.112 ± 0.007	0.975 ± 0.135
Liver	0.443 ± 0.630	75.655 ± 14.376
Sal. Gland	0.007 ± 0.005	0.605 ± 0.156
Fat	0.004 ± 0.003	0.327 ± 0.143
Intestine	0.015 ± 0.003	0.932 ± 0.149
Muscle	0.002 ± 0.001	0.103 ± 0.023
Skin	0.007 ± 0.003	0.685 ± 0.718
Lung	0.152 ± 0.254	25.655 ± 11.674
Spleen	0.342 ± 0.473	45.252 ± 4.626
Kidney	0.221 ± 0.104	6.032 ± 1.007
Blood	0.014 ± 0.005	0.698 ± 0.115
Thyroid	0.032 ± 0.033	5.838 ± 4.368
Urine	0.563 ± 0.350	41.202 ± 9.209
Tumor	164.817 ± 101.445	0.538 ± 0.219

The therapeutic intra-tumor protocols applied on Group A results in a statistically significant, time-dependent decrease of tumor volume (Figure 3B). The consequence of the LB accumulation in the tumor mass leads to a prolonged therapeutic action, confirmed by a delay in tumor growth. On the contrary, treatment with the same protein concentration of LB missing of the pro-apoptotic domain BH3 (LBΔBH_3_), induces an exponential growth of cells leading to a rapid development of the tumor, almost identical to that observed in untreated (Ctrl) mice (Figure 3B) (*p* < 0.01, LB *vs.* Ctrl and *vs.* LBΔBH3). Consequently, LB treatment induces a significant increase in life span compared to Ctrl and LBΔBH_3_-treated mice (*p* < 0.05, LB *vs.* Ctrl and *vs.* LBΔBH_3_) with a total tumor regression in more than 40% of mice 70 days after tumor challenge (Figure 3C). However, liposome-based drugs are usually injected systemically in preclinical and clinic settings. The delivery of the liposomal “drugs” to the neoplastic site is mostly mediated by the EPR effect, a passive tumor accumulation of about 100 nm diameter particles due to the irregular and non-homogeneous tumor vascularization [11]. Here, in an attempt to increase LB stability *in vivo* and, consequently, to likely enhance tumor targeting and accumulation, the liposomal formulation was intentionally enriched with cholesterol (20%). Indeed, it was shown that an increase in membrane rigidity impedes the lipids extraction and the breakdown of proteoliposomes, associated with the activity of High Density Lipoproteins (HDL) in the blood [38]. Furthermore, this lipid composition might likely increase LB penetration through the BBB in a more clinical relevant animal model of GBM [39]. Compared to the intra-tumor administration of LB above described, the intravenous therapeutic protocol was slightly modified in order to avoid animal stress and possible infections at the site of injections. Mice were indeed treated every two days for five times and, as already reported, the one-day interval between injections should avoid the well-known Accelerated Blood Clearance (ABC), a process that decreases dramatically the blood residency of vesicles [40]. Although mice received a lower amount of LB compared to the intra-tumor protocol and despite a poor LB accumulation in the tumor (Table 1) the intravenous injection of LB show excellent therapeutic effects, validated by a statistically significant delay in tumor growth (*p* < 0.001, LB *vs.* Ctrl and *vs.* LBΔBH3), and followed by a significant increase in life span compared to untreated mice, or those treated with LBΔBH_3_ (Figure 4B,C) (*p* < 0.05, LB *vs.* Ctrl and *vs.* LBΔBH3). Furthermore, total tumor regression was observed in about 10% of LB-treated mice, 70 days after tumor challenge. In addition, no loss in body condition, that could interfere with eating or impairs ambulation or other parameters, is observed in LB-treated mice. Investigations at single organs level on the effects of liposomes injected intravenously will be necessary. However, since the treatment with LBΔBH_3_ does not exert any anti-tumor effect, the encouraging animal outcome obtained highlights the therapeutic potential of LB in a complex 3D organized GBM in a living system.

### 2.4. Apoptosis on Isolated Tumors: TUNEL Assay

**Figure 5:**
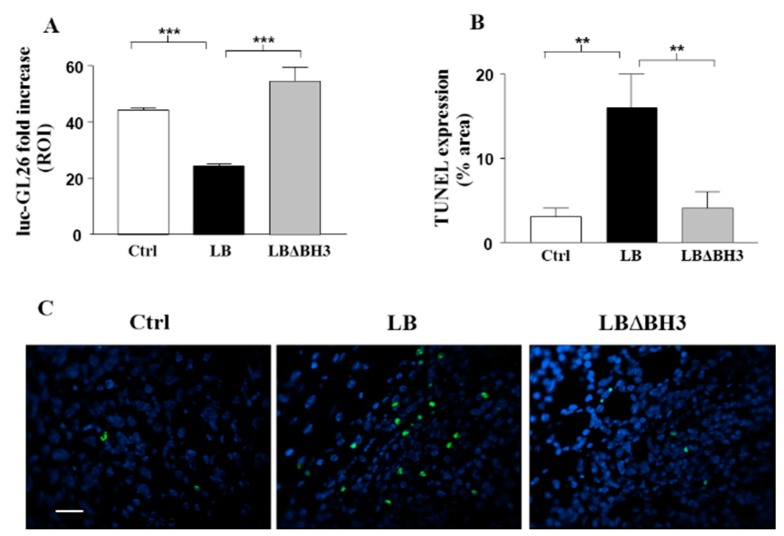
Bak Liposomes (LB) anti-tumor effects correlate with increased TUNEL staining in glioblastoma cells. (**A**) Tumor expansion over time in response to intravenous LB and LBΔBH_3_ administration evaluated by bioluminescence imaging (BLI) in luc-GL26-bearing mice (*n* = 5/group). Columns represent, in region of interest (ROI), the ratio between the tumor size at the first day of the treatment and the size of the tumor one week after the last treatment. *** *p* < 0.001, LB *vs.* Ctrl and *vs.* LBΔBH_3_; (**B**) Evaluation of TUNEL staining on tumors removed from mice (*n* = 3/group). ** *p* < 0.01, LB *vs.* Ctrl and *vs.* LBΔBH_3_; (**C**) Representative fluorescent images of TUNEL staining. Bar: 50 µm.

In a last set of experiments, luc-GL26 cells were subcutaneously inoculated in the flank of mice and treated as in Group B. Tumor growth was monitored by bioluminescence imaging (BLI), once a week for three weeks (Day 20, 27 and 34) after implantation. The treatment with LB proves to be very efficient, leading to a significant inhibition of tumor growth at Day 34 (Figure 5A) (*p* < 0.001, LB *vs.* Ctrl and *vs.* LBΔBH_3_). At the same time point, as shown in Figure 5B, LB treatment leads to a significant increase in the level of TUNEL-positive cells when compared with tumor cells derived from Ctrl or LBΔBH_3_-treated mice (*p* < 0.01, LB *vs.* Ctrl and *vs.* LBΔBH_3_). In Figure 5C, a representative field of each tumor shows an absence of apoptotic nuclei in samples derived from Ctrl and LBΔBH_3_-treated mice, whereas, green spots highlight the cellular apoptotic death process in the LB-treated tumor. This TUNEL assay performed one week after the end of treatments showed that LB injected through the blood stream induce tumor cell apoptosis, confirming results previously obtained in colon cancer cells [17].

## 3. Experimental Section

### 3.1. Cell-Free Production and Purification of Proteoliposomes

Human Bak was produced using a Rapid Translation System (RTS 500, Roche Diagnostics) according to the instruction manual and following the optimized protocol for membrane proteins, described previously [21]. BakΔBH3 (Bak missing the pro-apoptotic domain BH3) was used as negative control.

Briefly, liposomes were prepared using the classical Bangham method from a mixture of DOPC (1,2-Dioleoyl-*sn*-Glycero-3-Phosphocholine), DOPE (1,2-Dilinoleoyl-*sn*-Glycero-3-Phosphoethanolamine), DMPA (1,2-Dimyristoyl-*sn*-Glycero-3 Phosphate) and cholesterol in the ratio of 40:20:20:20. Liposomes were added directly to the RTS reaction mixture to produce LB in a one-step reaction. Purification of LB (or LBΔBH3) was performed by ultracentrifugation onto a discontinuous sucrose gradient, as described [21]. Recovered fractions were then loaded onto a 15% polyacrylamide gel, and fractions purity was checked by Coomassie blue staining. The presence of Bak monomers and oligomers was confirmed by Western blotting using a monoclonal anti-His HRP-conjugated antibody and quantified as described [21]. For the biodistribution study, LB were produced by Synthelis SAS using an exclusive UJF patented cell-free system.

### 3.2. Cell Line and Treatments

GL26 mouse glioblastoma cells were cultured in DMEM medium supplemented with 10% fetal bovine serum and Penicillin-Streptomycin (10,000 U/mL) (Cambrex Bio Science, Walkersville, MD, USA) at 37 °C in a humidified 5% CO_2_ incubator. For *in vivo* experiments, cells were also transduced with a retrovirus for the constitutive expression of the firefly luciferase gene, providing the luc-GL26 cell line. Luciferase activity was visualized by bioluminescence imaging (BLI; IVIS Caliper Life Sciences, Hopkinton, MA, USA) after 10 min incubation with 150 μg/mL of D-luciferin (Caliper Life Sciences), as described previously [41].

For the cell viability test, GL26 cells were seeded in a 96-well plate (1 × 10^5^ cells/well) and treated for 24 h with (i) LB (0.14, 0.35 and 1.07 μM corresponding to 2, 5, 10 µg/10^6^ cells); (ii) Etoposide (Eto, 25 µM; Sigma-Aldrich, St. Louis, MO, USA) or Cisplatin (CDDP, 10 µM), as positive controls. Empty liposomes in Phosphate-Buffered Saline (PBS), were used as negative control. MTT was then added to each well (100 µg) and cells incubated 5 h at 37 °C. To solubilize blue crystals, DMSO (200 µL/well) was added and the enzymatic activity was quantified by a multi-well scanning spectrophotometer (Multiscan Spectrum, Thermo Labsystem, Beverly, MA, USA).

To investigate, by Western Blot analysis, the molecular signaling activated by LB treatment, 1 × 10^6^ GL26 cells were cultured in 6-well plates and treated with (i) 1.07 μM purified LB; (ii) 250 nM Bortezomib (BZM; Velcade; Janssen-Cilag International NV, Beerse, Belgium); (iii) 25 μM Eto; (iv) 10 μM Staurosporine (Stauro; Sigma); and (v) 250 nM U0126 (Calbiochem, San Diego, CA, USA) and incubated for 6, 12, and 24 h before lysing.

After treatments, cells were washed in Dulbecco’s PBS incubated for 10 min on ice in 100 μL of RIPA Buffer (20 mM Tris/HCl pH 7.4, 150 mM NaCl, 1% Nonidet P-40, 0.1% Na-deoxycholate, 1 mM EDTA) supplemented with protease inhibitors (Complete Protease Cocktail, Roche, Indianapolis, IN, USA), 1 mM Na_3_VO_4_, and with 1 mM PMSF. Cells were centrifuged, the pellets discarded and protein concentration determined by a colorimetric assay (BCA Assay, Pierce, Rockford, IL, USA). Lysates (50 μg) were loaded and proteins separated by SDS-polyacrylamide gel (12%–15%) electrophoresis and transferred to nitrocellulose membranes (Bio-Rad, Hercules, CA, USA). Blots were blocked in 5% milk TBS-T for 3 h at room temperature. All primary antibodies were diluted in TBS-T, 5 mg/mL BSA: rabbit anti-pERK1/2 and rabbit anti-JNK (Cell Signaling technology, Inc., South San Francisco, CA, USA) 1:1000; rabbit anti-Bax (Calbiochem) 1:1000; mouse anti-α tubulin (Sigma) 1:5000. Mcl-1 detection (mouse anti-Mcl-1, 1:500; BD Biosciences Pharmigen) was performed on isolated mitochondria from 20 × 10^6^ GL26 cells using the Mitochondria Isolation Kit (Pierce, Rockford, IL, USA). Cytoplasmic fractions were prepared starting from 2 × 10^6^ GL26 cells using the NE-PERTM Nuclear and Cytoplasmic Extraction Kit (Pierce, Rockford, IL, USA). The peroxidase-conjugated secondary anti-mouse and anti-rabbit antibodies (Amersham Corp., Arlington Heights, IL, USA) were diluted 1:5000 in TBS-T. Immunoreactive bands were detected using the Lumi-Light chemiluminescence kit (ECL, Amersham, Buckinghamshire, UK).

### 3.3. Animal Model

Mice were purchased from Charles River Laboratories (Cambridge, MA, USA) and housed under specific pathogen-free conditions. Animal experiments were performed in accordance with the EU directives approved by the Institutional Animal Care and Use Committee of Grenoble University (UJF), or by the licensing and ethical committee of the National Cancer Research Institute, Genoa, Italy, and by the Italian Ministry of Health.

Six week-old, C57BL/6JRj female mice were injected subcutaneously with GL26 cells and treated either intra-tumor (*i.t.*; Group A) or intravenous (*i.v.*; Group B) with LB. Tumor volumes were calculated by the formula 4/3 π*abc*, where *a*, *b* and *c* represent the three diameters of the tumor mass. 

### 3.4. Biodistribution Analysis

The presence of poly-histidine tail (His-Tag) on the Bak protein construct allowed a site-specific labeling of LB with radioactive Technicium (^99m^Tc), using a commercially available kit (Isolink^®^ kit; Mallinckrodt Pharmaceuticals, Dublin, UK). Pertechneate (VII) ion (^99m^TcO4^−^) was eluted from a ^99^Mo/^99m^Tc generator. The radiochemical purity was determined by Thin Layer Chromatography in acetonitrile/water as mobile phase.

To determine the best evaluation time for the biodistibution study, in a Single Photon Emission Computed Tomography (SPECT/CT) dynamic imaging study, one mouse from both groups (A and B) were injected with ^99m^Tc-LB. At 1, 4 and 24 h after injection, both mice were anesthetized using isoflurane in a 1:1 mixture of room air and oxygen (3% for induction, 1.5%–2% for maintenance). Whole-body SPECT/CT images were acquired using a small animal dedicated gamma-camera (NanoSPECT/CT, Bioscan, Paris, France) equipped with four heads and high sensitivity/high resolution 1.4 mm multi-pinholes collimators. The reconstruction and quantification of the SPECT/CT images were performed using dedicated software (InvivoScope). The results obtained allowed us to choose 24 h following *i.t.* injection (Group A) and 4 h following *i.v.* injection (Group B) as the optimum time for organ collection and radioactive measurements.

Thus, tumor-bearing mice (*n* = 2/group) received *i.t.* or *i.v.* injections of ^99m^Tc-LB (12.7 ± 1.6 and 4.6 ± 1.9 MBq, respectively); samples of tissues and fluids were harvested at the chosen times, and weighted. Radioactivity was determined by a gamma-well counter (Cobra II, Packard) using a 112–168 keV energy window. Counts were corrected for background and decay. The results (mean ± S.D.) are expressed as percent injected dose per gram of tissue (%ID/g).

### 3.5. In Vivo Therapeutic Studies

Group A mice were subcutaneously inoculated into the rear left flanks with 1 × 10^5^ GL26 cells in 100 µL of PBS and examined daily for tumor growth. When tumor reached 150 mm^3^ (12 days post cells injection), mice were randomly divided into three groups and treated *i.t.* with 75 µg LB (*n* = 7) or LBΔBH_3_ (*n* = 7) as negative control, in 100 µL PBS, every day for seven days. Control mice (*n* = 5) received PBS.

Group B mice were subcutaneously inoculated with 1.2 × 10^5^ of GL26 or luc-GL26 cells (6 and 8 mice/group, respectively). When the tumor mass reached a size of about 50 mm^3^ (10 days post cells injection), mice were treated *i.v*. every two days for five times total with LB or LBΔBH_3_, as above. Control mice received PBS.

In each experiment, when tumor size reached about 1000 mm^3^, mice were sacrificed by cervical dislocation after being anesthetized with xilezine (2% Xilor, Bio98 Srl, Milan, Italy). Experiments were performed twice with similar results.

### 3.6. TUNEL Assay on Tumor Sections

Terminal deoxynucleotidyl transferase dUTP nick end labeling (TUNEL) is a common method for detecting DNA fragmentation that results from apoptotic signaling cascades. To evaluate the *in vivo* pro-apoptotic effects of LB, 3 out of 8 luc-GL26-bearing mice/group (Group B) were randomly sacrificed one week after the end of treatments. Tumors were collected and processed for paraffin-embedding, as described [42]. TUNEL assay was performed using a commercially available apoptosis detection kit (*In situ* Cell Death Detection, POD; Roche Molecular Biochemicals, Mannheim, Germany), as described [42].

### 3.7. Statistics

All the analyses were performed with Prism 5 software (GraphPad, La Jolla, CA, USA). The results are expressed as mean ± standard deviation (S.D.). Two-way analysis of variance (ANOVA), followed by Tukey’s Multiple Comparison Test, was used to evaluate differences within treatments, survival curves were drawn as Kaplan–Meier Cumulative Proportion Surviving graphs, and corresponding *p*-values were calculated by the use of the log-rank (Chi-square) test. Asterisks indicate the following *p*-value ranges: * *p* < 0.05, ** *p* < 0.01, *** *p* < 0.001.

## 4. Conclusions

This study presents evidence that cell-free technology is a powerful method for producing therapeutic LB in a one-step reaction. *In vitro* experiments on GBM cells confirm that LB are able to exert strong cytotoxic effects. Results on a GBM mouse model show potent anti-tumor effects independently from the LB route of administration performed. Future directions will be focused in further improving the *in vivo* stability of LB (*i.e.*, by adding PEG to the liposomal formulation), and to increase tumor targeting and accumulation of LB into the tumor site by decorating liposomes with specific (GMB-recognizing/tumor vasculature-recognizing) ligands [43]. This “second generation” of LB would represent an efficient, pro-apoptotic therapy to treat GBM.
